# BSDA: Bayesian Random Semantic Data Augmentation for Medical Image Classification

**DOI:** 10.3390/s24237511

**Published:** 2024-11-25

**Authors:** Yaoyao Zhu, Xiuding Cai, Xueyao Wang, Xiaoqing Chen, Zhongliang Fu, Yu Yao

**Affiliations:** 1 Chengdu Institute of Computer Application, Chinese Academy of Sciences, Chengdu 610213, China; zhuyaoyao19@mails.ucas.ac.cn (Y.Z.); caixiuding20@mails.ucas.ac.cn (X.C.); wangxueyao221@mails.ucas.ac.cn (X.W.); chenxiaoqing@casit.com.cn (X.C.); fzliang@casit.com.cn (Z.F.); 2 The School of Computer Science and Technology, University of Chinese Academy of Sciences, Beijing 101408, China

**Keywords:** semantic data augmentation, medical image, variational Bayesian

## Abstract

Data augmentation is a crucial regularization technique for deep neural networks, particularly in medical imaging tasks with limited data. Deep learning models are highly effective at linearizing features, enabling the alteration of feature semantics through the shifting of latent space representations—an approach known as semantic data augmentation (SDA). The paradigm of SDA involves shifting features in a specified direction. Current SDA methods typically sample the amount of shifting from a Gaussian distribution or the sample variance. However, excessive shifting can lead to changes in data labels, which may negatively impact model performance. To address this issue, we propose a computationally efficient method called Bayesian Random Semantic Data Augmentation (BSDA). BSDA can be seamlessly integrated as a plug-and-play component into any neural network. Our experiments demonstrate that BSDA outperforms competitive methods and is suitable for both 2D and 3D medical image datasets, as well as most medical imaging modalities. Additionally, BSDA is compatible with mainstream neural network models and enhances baseline performance. The code is available online.

## 1. Introduction

Deep learning methods can assist clinicians in rapid examination and accurate diagnosis [1,2]. However, these methods are data-demanding, and medical images are often scarce. For example, insufficient patients with specific diseases or a lack of medical equipment can lead to biased, overfitting, and inaccurate models [1]. Data augmentation (DA) is a common regularization technique to address these issues [3,4]. The variety of medical image modalities (e.g., MR, CT, X-ray, Retina) and their association with different clinical diseases necessitate specialized knowledge and careful debugging for image-level data augmentation methods to significantly improve network performance. Although automated DA methods [5,6] can avoid manual debugging, they are computationally expensive. Additionally, image-level DA methods often need help to improve sample diversity and achieve semantic transformations. While generative DA methods [7,8,9,10] can enhance semantic diversity, they are also computationally expensive and complex to train, making them challenging for medical practitioners without a computer science background.

Upchurch et al. [11] demonstrated that straightforward linear interpolations within feature space can achieve significant image transformations, effectively enabling a broader representation of variations within each class. From a theoretical perspective, Rajput et al. [12] create an ϵ-net within the feature space that not only introduces additional variability but also strengthens classification boundaries by expanding the feasible decision regions. Liu and Mirzasoleiman [13] further explored this by examining additive noise-based data augmentation, showing that the process amplifies specific smaller singular values of the Jacobian matrix, enhancing both learning efficacy and model generalization. Recent studies have shown that semantic data augmentation (SDA) can enhance network performance [14,15,16,17]. The ISDA method [18], which minimizes an upper bound of the expected cross-entropy loss on the augmented dataset, stands out in this domain. Specifically, ISDA facilitates implicit DA by translating deep features along semantic directions in the feature space, creating new representations that preserve class identity but offer varied semantic information. In detail, the deep feature space harbors various semantic directions, and translating features along these directions yields new sample features with identical class identities but altered semantics [18].

**Figure 1:**
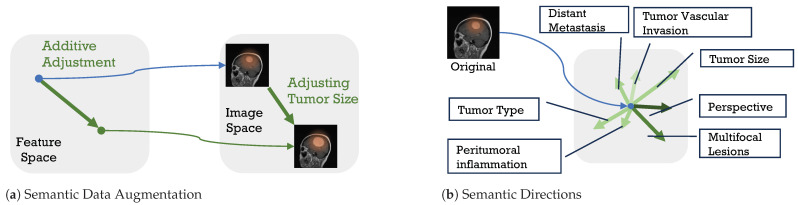
Example of semantic data augmentation. (**b**) is a non-exact example of different semantic directions in the feature space along which moving can change the semantics. (**a**) shows a non-exact example of semantic data augmentation in feature space.

**Figure 2:**
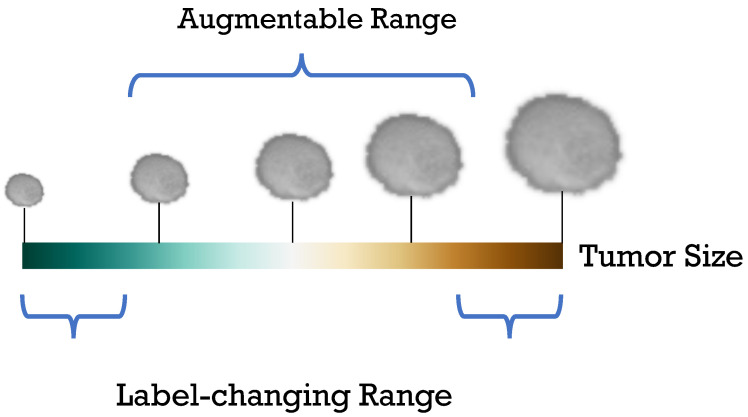
Imprecise example of augmentable semantic magnitude. For instance, in the tumor grading task, if the centermost value (white) is an input sample, the curly brackets indicate the range that does not change the original label, and the augmented samples beyond that range change the original label.

For instance, translating features along dimensions representing “tumor size” in a deep feature space yields variations in this attribute across generated samples without altering other defining characteristics. This process is visualized in Figure 1; Figure 1b illustrates various semantic directions in the feature space, while subfigure Figure 1a shows a transformation along the “tumor size” axis, producing a modified image with adjusted tumor size. This approach enriches the dataset’s diversity, offering more comprehensive semantic coverage that enhances training robustness and model generalizability. Unlike traditional methods that modify images directly, this approach generates new data at the feature level, such as operating random disturbances, interpolations, or extrapolations within the feature space for augmentation [19]. Generally, SDA methods are considered as a translation of the feature space [17,18,19,20], which implies the existence of two critical parameters: the magnitude of translation and direction. Current SDA methods [17,18] mainly aim to improve the direction, whereas the magnitude of translation should be equally important because over-translation may violate the image label. While some SDA methods [19,20] sample translations from the distribution specified by the implementation, they risk getting caught up in changing labels. Figure 2 shows an example of alterations beyond the augmentable range within the category that may result in label changes.

To address this issue, we reconsider the paradigm of semantic data augmentation for medical image classification. Inspired by the automatic augmentation method RandAugment (RA) [6], we define a semantic data augmentation strategy that translates features in a specified direction with a specified magnitude, which includes two key hyperparameters: semantic magnitude and semantic direction. For semantic magnitude, we treat the augmentable (label-preserving) semantic magnitude as a random variable and estimate its distribution using variational Bayesian methods. And for semantic directions, similar to the image space augmentation approach [21], it is a naive idea to select semantic directions randomly, but it does not make sense to perform augmentation in certain directions [18], a view based on image space. Some DA approaches [22,23] are adequate for downstream tasks, although they do not make sense for vision, and the quantitative evaluation approaches proposed in [24] explain why vision-meaningless DA approaches are still practical. Not coincidentally, Ref. [17] points out that adding a random Gaussian perturbation to the features significantly improves the Empirical Risk Minimization (ERM), although it does not follow any meaningful direction. Even the perturbation of randomness due to the reparameterization introduced by variational inference benefits the network’s learning of features [17]. Therefore, we do not augment all directions but randomly select semantic directions like the random selection transform in image space.

**Figure 3:**
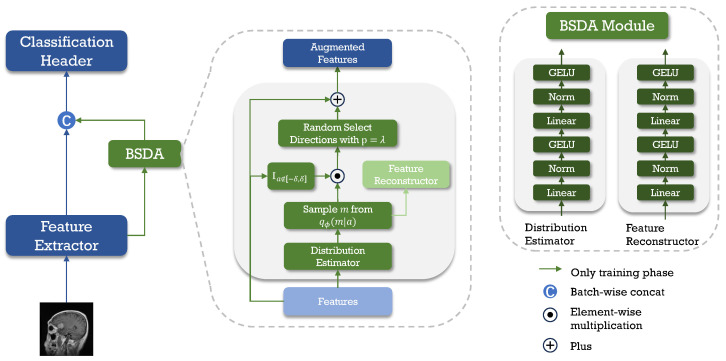
The framework of BSDA as a plug-in for the neural network.

In summary, we propose a simple yet efficient feature-level method for semantic data augmentation called Bayesian Random Semantic Data Augmentation (BSDA). BSDA adds semantic magnitudes to randomly selected directions, with the magnitudes obtained by sampling from augmentable semantic magnitude distribution estimated using variational inference. Figure 3 shows BSDA inserted into the network as a plug-in. Our main contributions are:

- We propose a high-performance Bayesian Random Semantic Data Augmentation plug-and-play module, BSDA, for medical image classification.
- We experimentally demonstrate that BSDA outperforms current data augmentation methods.
- We provide experimental evidence demonstrating that our module is applicable to images of different dimensions, various modalities, and diverse network architectures.

## 2. Related Work

### 2.1. Data Augmentation

In image recognition tasks, data augmentation techniques like random flipping, panning, and rotation make the network learn certain invariants [21,25]. These methods rely heavily on empirical knowledge, and a specific augmentation strategy can only be used for a particular dataset. AutoAugment [5] was the first technique to perform automatic augmentation by searching for a better strategy across numerous solution spaces using reinforcement learning. However, due to the large size of its search space, other works (e.g., RA [6], DADA [26]) have also shown strong performance by improving the search algorithm. While automated data augmentation algorithms have shown strong capabilities, the training of deep neural networks remains computationally intensive. Image Erasure [27] and Image Mixing [22,23,28] enhance the performance of the network by performing random erasure or mixing of images in the image space. Generative models have also shown strong performance in image generation, with some works [29,30] developing data augmentation techniques based on image generation. However, these methods require additional training of a generative model for each dataset. A Bayesian data augmentation method proposed in [31] learns the distribution of features from the training set by generating an adversarial network implementing generalized Monte Carlo expectation maximization and samples from it to obtain augmented data. It is worth noting that our method is similar in name but differs in approach. Several studies have explored the efficiency of data augmentation. Ref. [13] notes that data augmentation modeled as additive perturbation improves network performance by amplifying and perturbing the singular values of the network’s Jacobian determinant. Similarly, Ref. [12] showed a lower bound on the data required for data augmentation, demonstrating that the general use of DA requires an exponential amount of data before it leads to larger bounds.

### 2.2. Semantic Data Augmentation

Semantic data augmentation is achieved by label-preserving translation in the semantic space, generating new sample points in the feature space. Three semantic augmentation schemes involve adding random Gaussian noise with mean 0 and variance $\sigma^{2}$ to the features, interpolating neighbors, and extrapolating [19]. Similarly, OnlineAugment [32] achieves excellent performance by using Gaussian noise to perturb features, but it tends to generate non-class-preserving sample points. Furthermore, Ref. [17] used a moving average to estimate the feature covariance matrix online during training and modeled the cross-feature joint noise distribution. ISDA [18] is a novel semantic data augmentation algorithm that enhances dataset diversity by translating training samples in the deep feature space along semantically meaningful directions, improving the generalization performance of deep models with minimal computational cost. A shape space-based feature augmentation method projects multiple image features into a pre-shape space. Ref. [33] uses Wasserstein geodesic interpolation to augment data and regularize performance. Moment Exchange is an implicit data augmentation method that enhances recognition models by replacing and interpolating learned features’ moments (mean and standard deviation) between training images. This forces models to extract training signals from these moments, thus improving generalization across benchmark datasets [34].

## 3. Method

In this section, we will formalize the SDA method in Section 3.2 and our proposed BSDA method in Section 3.3.

### 3.1. Preliminary

Consider training a deep neural network $G_{\Theta }=f_{1}\circ f_{2}$ includes two parts: a feature extraction network $f_{1}$ and a classification network $f_{2}$ with parameters Θ on a dataset $D={\left\{\left(x_{i},y_{i}\right)\right\}}_{i=1}^{N}$, where each $y_{i}$ represents a label belonging to one of *c* classes. The output of $f_{1}$ is a *k* dimensions feature vector $a=f_{1}\left(x\right)\in R^{k}$, which is then input into $f_{2}$ to predict the target $\hat{y}=f_{2}\left(a\right)=f_{2}\left(f_{1}\left(x\right)\right)$, where $\hat{y}$ is the predicted class label. We refer to a as the original feature vector for clarity.

### 3.2. Semantic Data Augmentation

Generally, semantic data augmentation (SDA) methods are considered a translation of the feature space and can be formalized as follows:

(1) $$\tilde{a}=a+d⊙m,$$

where $\tilde{a}$ is an augmented feature. $d\in {\left\{0,1\right\}}^{k}$ is a binary vector for randomly selected semantic directions, and ⊙ denotes element-wise multiplication. $m\in R^{k}$ represents the semantic magnitude. Furthermore, to preserve specific properties of the features, such as low rank [35,36,37], we mask semantic directions corresponding to zero feature values, and we have:

(2) $$\tilde{a}=a+I_{a\neq 0}d_{\lambda }⊙m,$$

where $I_{a\neq 0}$ is an indicator function when a≠0, and the value of this function is 1.

### 3.3. Bayesian Random Semantic Data Augmentation

Each SDA method defines the variables in Equation (1) differently. Bayesian Random Semantic Data Augmentation (BSDA) defines the semantic direction d as a random variable that obeys a Bernoulli distribution with parameter λ for each dimension, denoted $d_{\lambda }$. And for semantic magnitude m, BSDA defines m∼p(m|a) as a random variable, where distribution p(m|a) means the range of augmentable features when given the original feature vector a. Unfortunately, the distribution p(m|a) is unknown.

#### 3.3.1. Estimate the Magnitude Distribution

To obtain p(m|a), we introduce a model $q_{\phi_{m}}\left(m|a\right)$ to approximate the true distribution p(m|a). The Kullback–Leibler (KL) divergence measures the similarity between these two distributions, aiming to make $q_{\phi_{m}}\left(m|a\right)$ closely match p(m|a) by maximizing the KL divergence. Thus, our optimization goal is as follows:

(3) $${\tilde{\phi }}_{m}=\underset{\phi_{m}}{\arg\max}D_{KL}(q_{\phi_{m}}\left(m|a\right)||p\left(m|a\right)).$$

Removing the terms that are not related to the parameter $\tilde{\phi_{m}}$ in $D_{KL}$, we have:

(4) $$\begin{array}{ccc} \; & D_{KL}(q_{\phi_{m}}\left(m|a\right)||p\left(m|a\right))\; & =\mathrm{KL}(q_{\phi_{m}}\left(m|a\right)||p\left(m\right))-E_{m∼q_{\phi_{m}}\left(m|a\right)}\left(\log p(a|m)\right). \end{array}$$

#### 3.3.2. Loss Function of BSDA

The first term of Equation (4) can be calculated easily. The second term estimates the features a given m, which is more challenging to compute. Drawing inspiration from the design of Variational Autoencoders (VAEs) [38], we introduce a reconstruction network for learning this term, rewriting the second term as $p_{\phi_{a}}\left(a|m\right)$. Then, we obtain the loss function of BSDA as follows:

(5) $$\begin{array}{ccc} \; & L_{B}\left(\phi_{m},\phi_{a};a\right)\; & =-\mathrm{KL}(q_{\phi_{m}}\left(m|a\right)||p\left(m\right))+E_{m∼q_{\phi_{m}}\left(m|a\right)}\left(\log p_{\phi_{a}}\left(a|m\right)\right). \end{array}$$

The second part of Equation (5) depends on the model, and we use MSE loss in BSDA. Assuming the marginal distribution p(m) follows a normal distribution N(0,I) and $q_{\phi_{m}}\left(m|a\right)$ also follows a normal distribution $N(0,\sigma^{2})$, setting the mean to zero because we aim to learn the offset relative to the original rather than the augmented feature. Thus, the loss function of BSDA is given by:

(6) $$\begin{array}{ccc} \; & L_{B}\left(\phi_{m},\phi_{a};a\right)=\; & -\frac{1}{2}\sum_{i=0}^{N}\left(1+\log\left(\sigma^{2}\right)-\sigma^{2}\right)+\frac{1}{2N}\sum_{l=1}^{N}{\left(\hat{a}-a\right)}^{2}, \end{array}$$

where $\sigma^{2}$ is estimated variance of BSDA, and $\hat{a}$ is a reconstructed feature using m.

#### 3.3.3. The Reparameterization Trick

We employ the reparameterization trick to facilitate the computation of the loss function while ensuring the gradient flow for effective backpropagation. The random variable m can be represented as the deterministic variable $m=g_{\phi_{m}}\left(\epsilon ,a\right)$, with ϵ∼N(0,I) being an auxiliary variable with an independent marginal distribution p(ϵ) and $g_{\phi_{m}}$ being a vector-valued function parameterized by $\phi_{m}$.

#### 3.3.4. Loss Function

Our augmentation method is training with $G_{\Theta }$. For convenience, we denote the loss of $G_{\Theta }$ as $L_{task}$, which typically uses cross-entropy loss in classification tasks. Therefore, the total loss function is:

(7) $$L=L_{task}^{a}+\alpha \left(L_{B}+L_{task}^{\tilde{a}}\right).$$

Different superscripts on $L_{task}$ distinguish between augmented and original features. The hyperparameter α is a dynamic value introduced to mitigate the impact of BSDA on the network during the initial stages of training when the network has yet to learn valuable features.

To summarize, the BSDA technique can be integrated into deep networks. We provide the pseudocode of BSDA in Algorithm 1.
**Algorithm 1**  The BSDA algorithm**Input: **DRandomly initialize Θ, $\phi_{a}$, and $\phi_{m}$**for** t=0 **to** *T*     Sample a mini-batch ${\left\{x_{i},y_{i}\right\}}_{i=1}^{B}$ from D;     Compute features $a_{i}=G\left(x_{i}\right)$;     Estimate variance of magnitude distribution $\sigma_{i}$;     Compute magnitude $m_{i}=\sigma_{i}⊙\epsilon_{i}$;     Compute *augmented feature ${\tilde{a}}_{i}$* according to Equation (2);     Compute reconstructed feature ${\hat{a}}_{i}$;     Compute L according to Equation (7);     Update Θ, $\phi_{a}$, and $\phi_{m}$;**end for**

## 4. Experiments

In this section, we first present the dataset in Section 4.1.1 and the training details in Section 4.1.2. Then, we evaluate the performance of BSDA in several dimensions, including the performance compared with SOTA models (Section 4.2), the performance for 3D images (Section 4.3), the performance and efficiency as a plug-in for different neural networks (Section 4.4), and the sensitivity of the parameters (Section 4.6), respectively. Finally, we perform an ablation study (Section 4.5) on BSDA and visualize the deep features (Section 4.7).

### 4.1. Datasets and Training Details

#### 4.1.1. Dataset

**Figure 4:**
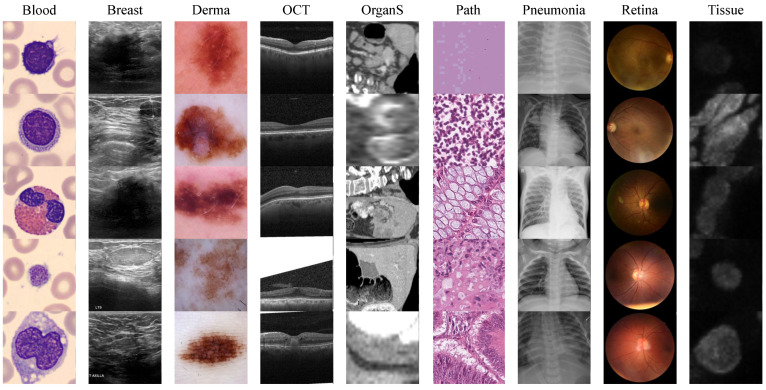
Samples of MedMNIST+. For convenience, we removed the MNIST suffix from the dataset. BloodMNIST consists of images of normal blood cells, while BreastMNIST features breast ultrasound images. DermaMNIST includes dermatoscopic images of pigmented skin lesions, and OCTMNIST presents optical coherence tomography images for retinal diseases. OrganS comprises 3D computed tomography images in various anatomical views. PathMNIST contains histology images from colorectal cancer tissue slides, and PneumoniaMNIST features pediatric chest X-ray images. RetinaMNIST consists of retina fundus images, whereas TissueMNIST includes images of kidney cortex tissue obtained through microscopy.

The MedMNIST+ dataset [39] is a widely used benchmark for medical images, including twelve preprocessed 2D and six preprocessed 3D datasets covering various medical imaging sources, representing essential biomedical imaging modalities such as X-ray, Optical Coherence Tomography (OCT), Ultrasound, CT, and Electron Microscopy. These datasets span a variety of classification tasks, including binary classification, multi-class classification, ordinal regression, and multi-label classification, with data scales ranging from 100 to 100,000 samples to accommodate diverse research requirements [39]. To ensure task consistency, all images were resized to 224 × 224 pixels for 2D and 64 × 64 × 64 voxels for 3D using cubic spline interpolation, preserving image integrity for model input. MedMNIST+ follows a standardized data split protocol, with all datasets divided into training, validation, and test sets in a 7:1:2 ratio at the patient level to maintain independence across sets. This combination of diverse imaging modalities, consistent preprocessing, and standardized splitting makes MedMNIST+ an excellent benchmark dataset for evaluating medical image classification models across various tasks, enhancing its relevance and utility for researchers in biomedical image analysis and machine learning. In this study, the selected nine 2D medical image datasets and five 3D medical image datasets in MedMNIST+ [39] covers twelve modalities, as shown in Figure 4. We further applied a series of data augmentation techniques to improve model generalizability and robustness. These included random rotations, flips, intensity adjustments, and elastic transformations to simulate common variations in medical imaging conditions, thus enhancing the model’s ability to learn invariant features. Please refer to Table 1 for a more detailed dataset overview.

#### 4.1.2. Training Details

Evaluation Protocols: We used the MedMNIST+ split training set and validation set to select hyperparameters and reported the results of the test set. The randomness arising from model selection is often ignored. For instance, does method A outperform method B only because the random search for A got lucky [40]? Therefore, we repeated the process three times with different random seeds. Every number we report is the mean of these repetitions and their estimated standard error. The area under the ROC curve (AUC) and accuracy (ACC) are used as the evaluation metrics.

Implementation Details: We implemented BSDA using PyTorch 2.2.2 and Torchvision 0.17.2 and experimented on an NVIDIA RTX 4090 GPU ×2 with an Intel 14900k CPU. During training, we utilized the AdamW [41] optimizer with an optimal learning rate of $1\times 10^{3}$, which was determined through a search among the values {$1\times 10^{3}$, $3\times 10^{4}$, $1\times 10^{4}$, $5\times 10^{5}$, $1\times 10^{5}$}. Additionally, we implemented a learning rate warm-up strategy for the first five epochs to enhance the training stability. The 2D image size is 224×224, and the 3D image size is 64×64×64. To ensure fairness, we maintained consistent training configurations across all experiments. The distribution estimator and reconstruction modules of BSDA consist of two fully connected layers, followed by BatchNorm and GeLU activation.

### 4.2. Comparison Experiment

First, we evaluated state-of-the-art methods on nine 2D medical image datasets in MedMNIST2D [39] (BloodMNIST, BreastMNIST, DermaMNIST, OCTMNIST, OrgansMNIST, PathMNIST, PneumoniaMNIST, RetinaMNIST, and TissueMNIST), which include a total of 513,429 samples. $\left(1\right)$ Cutout [42] is a data augmentation technique where parts of images are randomly masked out during training to improve model robustness and prevent overfitting. $\left(2\right)$ Mixup [28] is a data augmentation method that generates new training samples by interpolating between random pairs of examples and their labels, promoting better generalization. $\left(3\right)$ CutMix [23] is a data augmentation approach that replaces random patches of an image with patches from another image while also adjusting the labels proportionally, enhancing training diversity. $\left(4\right)$ ISDA [18] is a regularization method for deep networks that enhances model robustness by generating semantically meaningful examples using semantic data augmentation. Table 2 and Table 3 present the ACC and AUC of different 2D medical image datasets with state-of-the-art methods, respectively.

#### 4.2.1. ACC Results of MedMNIST2D+

Table 2 reveals that BSDA is the top-performing method, achieving the highest average accuracy of 81.9% across all evaluated datasets. ISDA is also an SDA method that performs strongly with an average accuracy of 81.0% but is slightly less consistent than BSDA. This demonstrates the advantages of semantic data augmentation methods for medical images and highlights that BSDA outperforms ISDA. For example, BSDA achieved 70.4% accuracy on TissueMNIST and 82.2% on OrgansMNIST, while ISDA achieved 68.1% and 80.8% on these datasets, respectively. Methods like CutMix [23], CutOut [42], and MixUp [28] offer comparable results, with average accuracies of 80.4%, 80.8%, and 80.1%, respectively, yet none consistently surpass the performance of ISDA and BSDA. RetinaMNIST is the most challenging dataset, with all methods showing lower accuracy levels around 50–53%, such as ISDA at 52.6% and BSDA at 53.3%. The results highlight the critical role of data augmentation techniques in enhancing model performance, as the baseline consistently underperforms with an average accuracy of 79.0%, emphasizing the necessity of employing sophisticated augmentation strategies in medical imaging tasks.

#### 4.2.2. AUC Results of MedMNIST2D+

The results of Table 3 reinforce the effectiveness of BSDA, which not only achieves the highest average AUC but also consistently performs across different datasets. BSDA achieves an average AUC of 93.9%, indicating its effectiveness in enhancing the model’s ability to distinguish between classes. This performance is slightly higher than other methods. BSDA exhibits lower variability in performance across most datasets, indicating more consistent results. For instance, its standard deviation in AUC is relatively low, especially compared to methods like Mixup [28] and ISDA [18], which show higher variability in datasets like PneumoniaMNIST and RetinaMNIST. ISDA and CutMix [23] also perform well but with higher variability. BSDA consistently outperforms other augmentation techniques in average accuracy (ACC) and area under the curve (AUC) across multiple MedMNIST datasets, demonstrating superior performance and reliability in medical image classification tasks.

### 4.3. Results of MedMNIST3D+

We selected five 3D MedMNIST datasets to validate the performance of BSDA. As shown in Table 4, BSDA enhances the performance of ResNet-18 across various 3D datasets, with improvements in both AUC and ACC for most datasets. For example, AdernalMNIST3D shows a substantial accuracy improvement from 71.5% to 89.8% and a slight AUC increase from 89.1% to 89.2%, suggesting that BSDA significantly enhances the model’s performance, particularly in accuracy. OrganMNIST3D shows an accuracy increase from 87.2% to 88.7% and an AUC improvement from 99.2% to 99.4%. This indicates that BSDA improves the correct classification rate and enhances the model’s ability to distinguish between classes. Similarly, NoduleMNIST3D experiences a rise in accuracy from 84.5% to 86.1% and in AUC from 87.9% to 89.2%, showing consistent improvements in both metrics. For FractureMNIST3D, accuracy rises from 53.5% to 55.0%, while AUC improves from 72.7% to 74.0%. These moderate improvements indicate that BSDA helps in better identifying fractures, though the dataset remains challenging. VesselMNIST3D also benefits, with accuracy increasing from 92.1% to 93.2% and AUC from 90.2% to 91.7%, reflecting improved precision and robustness in vessel identification. These results underscore the value of data augmentation techniques in improving both the discriminative power and the accuracy of deep learning models in medical imaging tasks, demonstrating BSDA’s effectiveness in enhancing model performance across diverse medical datasets.

### 4.4. Applicability of BSDA

We selected several mainstream architectures in computer vision to conduct experiments to verify the performance of BSDA combined with different models, including ResNet [21], EfficientNet [25], DenseNet [43], TinyViT [44], ViT [45], Swin Transformer [46]. Table 5 shows that the BSDA technique significantly improves accuracy (ACC) and area under the curve (AUC) across different convolutional neural networks, particularly on the DenseNet-121, ViT, and Swin Transformer series. For example, the accuracy of DenseNet-121 improved from 84.9% to 89.4%, and the AUC increased from 96.6% to 96.9%. In most cases, BSDA introduces a small additional time cost, up to 7.3% for EfficientNet-B0. Although BSDA leads to slight performance degradation on some networks (e.g., ResNet-50), overall, it improves model performance with manageable additional computational costs. Therefore, BSDA is a technique worth considering for improving model performance.

We analyzed the effectiveness of BSDA components and compared BSDA by adding random standard Gaussian noise to the latent space features. Table 6 indicates that random noise can somewhat improve model performance, but the improvement is limited. The BSDA model, with the introduction of the indicator function (Equation (2)) and reconstructor components, shows significant improvement over the baseline model, especially in terms of accuracy. Both the indicator function and reconstructor components are essential for BSDA. Notably, model performance degrades considerably when the indicator function and reconstructor components are removed. This is equivalent to eliminating the reconstructor, as well as the second term in Equation (5), which is comparable to simply making $q_{\phi_{m}}\left(m|a\right)$ similar to the standard Gaussian distribution. The model performance should be comparable to that of sampling noise from a standard Gaussian distribution added to the original features, because it is simultaneously constrained by the loss of the task, thus further making the samples sampled by the model $q_{\phi_{m}}\left(m|a\right)$ more favorable to the task.

### 4.5. Ablation Study

### 4.6. Sensitivity Analysis

To better understand the BSDA method, we conducted a series of experiments analyzing it from different perspectives. The following sections provide a detailed analysis of the results, including hyperparameter sensitivity analysis and loss weight analysis.

**Figure 5:**
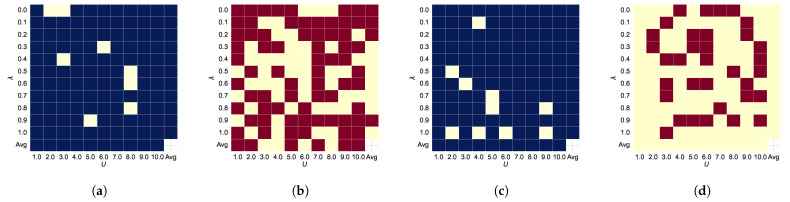
Ablation study for BSDA on BreastMNIST dataset using ResNet-18. Under the same hyper-parameters, if BSDA improves performance relative to the baseline (AUC = 89.6%, ACC = 81.0%), the heatmap displays red (AUC) or blue (ACC). In each subplot, the horizontal axis represents the sampling rate *U* of BSDA, while the vertical axis denotes the probability λ of random direction selection. (**a**,**b**) adds original features relative to (**c**,**d**). (**a**) ACC with original features. (**b**) AUC with original features. (**c**) ACC without original features. (**d**) AUC without original features.

**Figure 6:**
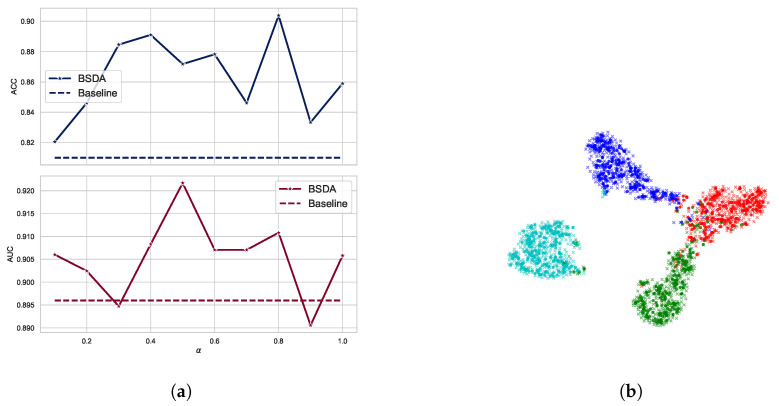
Sensitivity analysis and feature visualization. (**a**) Sensitivity analysis was conducted on the parameter α of BSDA using ResNet-18 on the BreastMNIST dataset. The horizontal axis indicates the values of α, while the vertical axis illustrates the AUC and ACC performance metrics. (**b**) Visualizing deep features using t-SNE on OCTMNIST, individual colors correspond to specific categories. Circular markers represent original features, while cross markers indicate augmented features generated by BSDA.

Hyperparameter Analysis: As shown in Figure 5a,b, Figure 6 show the AUC and ACC results of the joint features for different hyperparameters λ and *U* compared to the baseline, with red or blue indicating improvements over the baseline. Comparing Figure 5a–d, we see that the joint features improve model performance in most cases.

Weight of Loss: We conducted a sensitivity analysis of the hyperparameter α of BSDA using ResNet-18 on the BreastMNIST dataset. The results are shown in Figure 6a. We chose the hyperparameter α values from 0.1 to 1. It can be observed that the AUC and ACC metrics are stable in almost all cases, indicating that the BSDA method is not sensitive to the hyperparameter α. The AUC value is slightly lower than the baseline at α=0.3 or α=0.9, and the ACC value is somewhat lower than the baseline at α=0.1 or α=0.9. Empirically, we recommend α=0.5 as the default parameter.

### 4.7. Visualization of Deep Features

We visualize the deep features using t-SNE [47] in Figure 6b. Circular markers represent the original features, while the cross markers indicate the augmented features generated by BSDA. The results show that the augmented features are distributed around the original features, indicating that BSDA generates augmented features that are close to the original ones. Figure 6b also shows that the original features wrap tightly around the newly generated features. Intuitively, classifiers learned from the augmented features will be farther from the original features.

**Figure 7:**
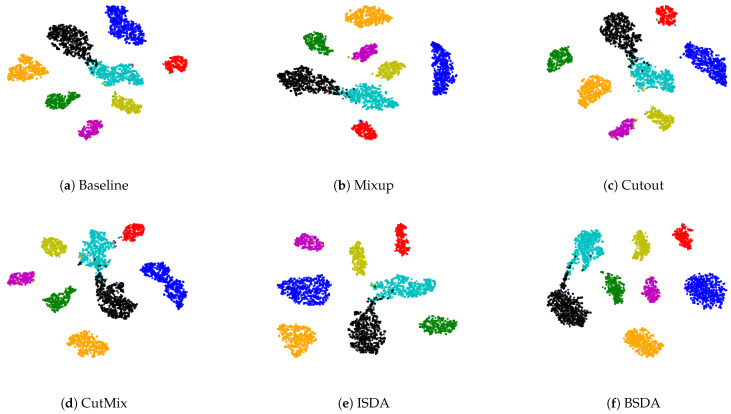
Visualizing deep features using t-SNE on BloodMNIST, individual colors correspond to specific categories. BSDA methods (Figure 7f) make the intra-class features more cohesive and more easily separable.

We also visualize the features learned by our method compared to other methods (without feature-level or image-level augmentation). Figure 7 shows that BSDA methods (Figure 7f) make the intra-class features more cohesive and more easily separable.

## 5. Discussion

### 5.1. Comparison with Other Methods

VAE: Our approach resembles VAE [38], where a latent variable is estimated and used for reconstruction. However, the relationship between our latent variable m and the inputs differs from VAE [38] and the latent variable representation of the inputs in VAE [38]. Our latent variable m represents the augmentable range in a given feature a without altering the label.

ISDA: Our approach differs from ISDA [18] in several key aspects: (a) it does not depend on the task’s loss function; (b) it serves as an implicit data augmentation method, complementing the explicit ISDA. This makes BSDA more convenient for special treatments in particular network branches. For instance, the BSDA module can be applied to both branches simultaneously in feature-decoupled networks, achieving post-decoupled feature enhancement.

### 5.2. Applications

BSDA, as an explicit data augmentation method, has many promising applications in medical imaging. For example, in the case of multimodal or multiparametric medical images, BSDA can be flexibly inserted into the network as a plug-in to provide semantic data augmentation for different modalities simultaneously, potentially enhancing the model’s performance. Another example is the multicenter setting, typical in medical imaging, where the model must be generalized to other domains. Some methods use feature decoupling-based or feature disentanglement-based domain generalization. BSDA can also provide semantic data augmentation for different decoupled branches, potentially enhancing the model’s generalization ability. Moreover, medical image datasets often need to be more balanced. Adding a balanced sampling strategy during data augmentation in the training phase can improve network performance on unbalanced datasets.

BSDA presents a versatile and efficient approach to data augmentation in medical imaging, promising significant improvements in model performance and generalization. Future work will optimize BSDA for specific medical imaging tasks and explore its potential in real-world clinical settings.

## 6. Conclusions

This paper introduces an efficient, plug-and-play Bayesian Random Semantic Data Augmentation (BSDA) method for medical image classification. BSDA generates new samples in feature space, making it more efficient and easy to implement. We experimentally demonstrate the effectiveness and efficiency of BSDA on various modalities, dimensional datasets, and networks.

## Figures and Tables

**Table 1:** An overview of datasets. We selected nine 2D medical image datasets and six 3D medical image datasets in MedMNIST+ covering twelve modalities. For convenience, we removed the MNIST suffix from the dataset.

Dataset	Data Modality	Tasks (Classes/Labels)	Samples	Training/Validation/Test
Blood	Blood Cell Microscope	Multi-Class (8)	17,092	11,959/1712/3421
Breast	Breast Ultrasound	Binary-Class (2)	780	546/78/156
Derma	Dermatoscope	Multi-Class (7)	10,015	7007/1003/2005
OCT	Retinal OCT	Multi-Class (4)	109,309	97,477/10,832/1000
OrganS	Abdominal CT	Multi-Class (11)	25,211	13,932/2452/8827
Path	Colon Pathology	Multi-Class (9)	107,180	89,996/10,004/7180
Pneumonia	Chest X-Ray	Binary-Class (2)	5856	4708/524/624
Retina	Fundus Camera	Ordinal Regression (5)	1600	1080/120/400
Tissue	Kidney Cortex Microscope	Multi-Class (8)	236,386	165,466/23,640/47,280
Organ3D	Abdominal CT	Multi-Class (11)	1742	971/161/610
Nodule3D	Chest CT	Binary-Class (2)	1633	1158/165/310
Adrenal3D	Abdominal CT	Binary-Class (2)	1584	1188/98/298
Fracture3D	Chest CT	Multi-Class (3)	1370	1027/103/240
Vessel3D	Brain MRA	Binary-Class (2)	1908	1335/191/382

**Table 2:** Comparing ACC% (accuracy) performance with state-of-the-art methods. We report mean values and standard deviations in three independent experiments. The best results are **bold-faced** and those underlined are second best. For convenience, we removed the MNIST suffix from the dataset. Official is the result reported in the MedMNIST+ [39], and Baseline is our implementation.

Method	Blood	Breast	Derma	OCT	OrganS	Path	Pne	Retina	Tissue	Avg
Official	95.8	83.3	75.4	76.3	77.8	90.9	86.4	49.3	68.1	78.1
Baseline	98.5±0.1	81.0±8.5	74.9±1.1	86.7±1.3	79.5±0.2	91.8±0.6	82.1±6.6	47.4±5.5	69.4±0.9	79.0
Mixup	98.4±0.2	83.5±3.2	$\underline{76.6\pm 0.9}$	89.5±0.8	80.5±0.8	89.5±2.4	81.6±6.1	51.3±0.9	70.4±0.5	80.1
Cutout	98.2±0.1	86.3±3.7	75.6±0.1	87.9±1.9	80.1±1.4	92.3±1.5	86.1±0.5	51.5±4.9	69.5±0.9	80.8
CutMix	98.3±0.1	84.6±0.6	76.3±0.5	87.5±0.3	80.6±0.5	90.3±2.3	83.6±7.5	52.2±1.5	$\underline{69.7\pm 0.0}$	80.4
ISDA	$\underline{98.7\pm 0.4}$	$\underline{86.1\pm 1.0}$	76.7±0.4	88.2±1.6	$\underline{80.8\pm 1.3}$	90.9±0.7	$\underline{87.2\pm 3.7}$	$\underline{52.6\pm 1.5}$	68.1±2.6	$\underline{81.0}$
**Our**	98.8±0.1	$\underline{86.1\pm 1.5}$	76.4±0.8	$\underline{88.8\pm 1.3}$	82.2±0.7	$\underline{91.9\pm 3.2}$	88.8±1.2	53.3±0.1	70.4±1.0	81.9

**Table 3:** Comparing the AUC% (area under the ROC curve) performance with state-of-the-art methods. We report mean values and standard deviations in three independent experiments. The best results are **bold-faced** and those underlined are second best. For convenience, we removed the MNIST suffix from the dataset. Official is the result reported in the MedMNIST+ [39], and Baseline is our implementation. Pne means Pneumonia dataset.

Method	Blood	Breast	Derma	OCT	OrganS	Path	Pne	Retina	Tissue	Avg
Official	99.8	89.1	92.0	95.8	97.4	98.9	95.6	71.0	93.3	92.5
Baseline	99.9±0.0	89.6±2.0	93.2±0.3	98.7±0.1	97.7±0.0	99.4±0.1	95.1±0.9	72.6±1.8	93.6±0.4	93.3
Mixup	99.9±0.0	89.5±1.2	92.7±0.5	98.7±0.3	97.8±0.2	98.7±0.4	95.8±0.4	71.9±1.3	93.9±0.1	93.2
Cutout	99.9±0.0	$\underline{91.1\pm 1.5}$	93.0±0.5	98.8±0.6	97.8±0.2	99.0±0.5	$\underline{95.9\pm 0.6}$	72.5±1.4	93.6±0.3	93.5
CutMix	99.9±0.0	90.7±1.0	92.9±0.4	$\underline{98.9\pm 0.2}$	$\underline{97.9\pm 0.1}$	99.0±0.5	96.4±0.6	73.4±1.3	93.9±0.0	$\underline{93.7}$
ISDA	99.9±0.0	89.3±2.0	93.0±0.4	99.0±0.3	97.8±0.3	99.4±0.1	95.0±1.1	$\underline{74.1\pm 1.4}$	93.6±0.4	93.5
**Our**	99.9±0.0	91.4±0.2	$\underline{93.1\pm 0.2}$	$\underline{98.9\pm 0.3}$	97.9±0.0	$\underline{99.2\pm 0.4}$	95.7±0.2	75.0±0.7	$\underline{93.7\pm 0.4}$	93.9

**Table 4:** Performance of different 3D datasets on the test set using ResNet-18 [21]. The better result is **bold-faced** compared with the Baseline. Official is the result reported in the MedMNIST+ [39], and Baseline is our implementation. For convenience, we removed the MNIST suffix from the dataset.

Dataset	ACC%			AUC%		
	Official	Baseline	Our	Official	Baseline	Our
Organ3D	90.7	87.2±0.7	88.7±1.4	99.6	99.2±0.1	99.4±0.1
Nodule3D	84.4	84.5±1.1	86.1±0.6	86.3	87.9±1.5	89.2±1.3
Adernal3D	72.1	71.5±2.3	83.8±3.6	82.7	89.1±2.7	89.2±3.4
Fracture3D	50.8	53.5±1.3	56.9±4.6	71.2	72.7±0.5	73.1±4.3
Vessel3D	87.7	92.1±1.1	93.2±0.3	87.4	90.2±5.5	91.7±5.8

**Table 5:** Evaluation of BSDA on different convolutional neural networks using the test set of PneumoniaMNIST [39]. The best results are **bold-faced** while the number in brackets denotes the performance improvements achieved by BSDA. The last column is about the additional time introduced by BSDA.

Network	ACC %		AUC %		Additional Time %
	Baseline	Our	Baseline	Our	
ResNet-18	82.1±6.6	88.8±1.2	95.1±0.9	95.7±0.2	3.7%
ResNet-50	87.0±3.5	86.3±3.4	96.8±0.7	96.9±0.2	5.9%
EfficientNet-B0	84.2±1.0	84.4±0.3	94.3±0.6	94.9±0.5	7.3%
DenseNet-121	84.9±3.3	89.4±2.6	96.6±0.3	96.9±0.9	1.5%
ViT-T	82.9±4.4	86.0±1.7	94.9±0.1	96.0±0.6	7.5%
ViT-S	81.1±5.9	87.2±1.8	95.3±0.6	95.9±0.6	5.8%
ViT-B	81.8±2.2	86.8±0.9	94.1±1.0	95.2±0.9	2.3%
Swin-T	73.6±11.4	77.0±4.1	87.3±4.7	92.0±0.2	1.4%
Swin-S	63.9±1.3	71.7±6.6	81.9±9.4	90.6±1.1	2.1%
Swin-B	62.5±0.0	62.5±0.0	88.3±1.9	88.3±2.5	1.3%

**Table 6:** Ablation study on OragnSMNIST. − means remove the corresponding module and + means add the module. Indicator is indicator function in Equation (2), Recon is reconstructor.

Setting	ACC%	AUC%
Base	79.5±0.2	97.7±0.0
BSDA	82.2±0.7	97.9±0.0
− Indicator	77.4±0.7	97.7±0.2
− Recon	76.8±1.8	97.6±0.2
− Indicator and Recon	80.2±0.4	97.8±0.1
Random Noise	79.6±0.3	97.9±0.1
+ Indicator	79.3±0.3	97.8±0.1

## Data Availability

The data are available online at https://medmnist.com/ (accessed on 26 January 2024).

## Abbreviations

The following abbreviations are used in this manuscript:

DA	Data Augmentation
SDA	Semantic Data Augmentation
BSDA	Bayesian Random Semantic Data Augmentation
RA	Randaugment
ISDA	Implicit Semantic Data Augmentation
AUC	Area Under the Curve
ACC	Accuracy
Blood	BloodMNIST
Breast	BreastMNIST
Derma	DermaMNIST
OCT	OCTMNIST
OragnS	OragnSMNIST
Path	PathMNIST
Pne	PneumoniaMNIST
Retina	RetinaMNIST
Tissue	TissueMNIST
Organ3D	Organ3DMNIST
Nodule3D	NoduleMNIST3D
Adernal3D	AdernalMNIST3D
Fracture3D	FractureMNIST3D
Vessel3D	VesselMNIST3D

## Appendix A. Additional Experiments

### Appendix Comparison with Data Augmentation Methods

To assess the effectiveness of our proposed method in classification tasks, we compare it against state-of-the-art data augmentation techniques within the context of medical image segmentation. All experiments are conducted under the same conditions as those established by MedAugment [3]. Table A1 summarizes the evaluation of BRSDA on the test set of the LUNG [48] dataset using ResNet-18 [21].

The results in Table A1 demonstrate that our method achieves the highest AUC and accuracy scores among the compared techniques, indicating its effectiveness in improving classification performance for medical image segmentation tasks. This superior performance highlights the potential of our approach in addressing the challenges posed by data scarcity and the need for effective augmentation strategies.

**Table A1:** Evaluation of BRSDA on the test set of LUNG [48] dataset using ResNet-18 [21] with state-of-the-art data augmentation methods. The best results are **bold-faced** and those underlined are second best. We report the results of different semantic data augmentation methods trained with non-semantic data augmentation methods.

Method	AUC%	ACC%
MedAugment [3]	92.20	78.01
AutoAugment [5]	89.25	82.98
AugMix [22]	87.61	75.18
Our	**93.03**	**85.11**
